# Small Wonders—The Use of Nanoparticles for Delivering Antigen

**DOI:** 10.3390/vaccines3030638

**Published:** 2015-08-10

**Authors:** Aya Taki, Peter Smooker

**Affiliations:** School of Applied Sciences, RMIT University, P.O. Box 71 Bundoora, Victoria 3083, Australia; E-Mail: aya.taki@rmit.edu.au

**Keywords:** nanoparticle, antigen delivery, templating system, adjuvant

## Abstract

Despite the discovery of many potential antigens for subunit vaccines, universal protection is often lacking due to the limitations of conventional delivery methods. Subunit vaccines primarily induce antibody-mediated humoral responses, whereas potent antigen-specific cellular responses are required for prevention against some pathogenic infections. Nanoparticles have been utilised in nanomedicine and are promising candidates for vaccine or drug delivery. Nanoparticle vehicles have been demonstrated to be efficiently taken up by dendritic cells and induce humoral and cellular responses. This review provides an overview of nanoparticle vaccine development; in particular, the preparation of nanoparticles using a templating technique is highlighted, which would alleviate some of the disadvantages of existing nanoparticles. We will also explore the cellular fate of nanoparticle vaccines. Nanoparticle-based antigen delivery systems have the potential to develop new generation vaccines against currently unpreventable infectious diseases.

## 1. Introduction

The fight to control infectious disease is an endless task for humanity. With millions of deaths from infectious disease each year, it has an enormous impact and is a burden on the global economy and health care system. Sanitation and prevention are key to reduce the impact of infectious disease [1], rather than drug treatments after infection has occurred. A number of infectious diseases have been prevented since the birth of vaccines by Jenner and Pasteur 200 years ago; however, there are still no registered or effective vaccines for some of the most prevalent diseases in the modern era. These infectious and parasitic diseases include acquired immune deficiency syndrome (AIDS), tuberculosis, malaria, leishmaniasis and hepatitis C, which cause millions of deaths every year (data shown in Table 1). Recently, an outbreak of the Ebola virus was also observed in the West African region (2014–2015), emphasizing the urgent need for an effective vaccine delivery system to prevent future epidemics.

**Table 1:** Number of deaths caused in 2013 by infectious and parasitic diseases for which effective vaccines are not yet available.

Disease	Number of Reported/Estimated Deaths	Number of Reported/Estimated Cases
AIDS (HIV)	1,590,952/-	-/35,000,000
Tuberculosis	-/1,072,678	5,725,317/11,468,559
Malaria ^1^	107,225/624,568	48,231,939/207,400,000
Hepatitis C	-/425,000	-/140,000,000
Leishmaniasis ^2^	-/25,000	213,871/-
Schistosomaiasis	23,313/-	-/300,000
Trypanosomasis ^3^	19,026 ^4^	6,314 newly reported/20,000
Ebola virus (2014–2015 outbreak) ^#^	11,080	26,759

^1^ 2012 data; ^2^ Cases of cutaneous leishmaniasis and visceral leishmaniasis combined; ^3^ Cases for *T.b. gambiense* and *T.b. rhodesiense* combined; ^4^ 2011 data; ^#^ Ebola outbreak record data up to 10th May 2015 WHO Ebola Situation report [2]. Latest data available from World Health Organization (WHO), Global health observatory data repository [3].

For a vaccine to be successful, it must satisfy a number of important criteria. Firstly, it must be able to elicit an immune response with a minimal number of doses (ideally a single dose), and provide long-lasting protection [4,5]. Secondly, it must be totally safe and effective in all vaccinated subjects, as the vaccine will be distributed across all age groups, including infants and children. It should also be stable and inexpensive to manufacture [5]. Further to these requirements, an effective antigen delivery system must be able to deliver antigens to specific cells that play a crucial role in immune initiation, and initiate a specific immune response accordingly. The efficiency of the delivery system must also not be diminished by pre-existing immunity [6], or induce immune tolerance.

Traditional vaccines using attenuated or killed organisms constitute the majority of vaccine formulations currently used in the market. DNA vaccines are capable of eliciting potent cellular responses [7], and four DNA vaccines are currently licensed for veterinary use. However, despite this success, the progress of DNA vaccines for human use has been slow. The inability to induce appropriate levels of immune response alone is a major hurdle for most DNA vaccines [8], although often strong CD8^+^ responses can be induced. Several strategies have been studied to improve the immunogenicity of DNA vaccines, including co-delivery of stimulatory molecules, gene optimisation, and delivery in an attenuated viral or bacterial vector (reviewed in [9]). Subunit vaccines offers a safe alternative to attenuated vaccines, and are currently used in several licensed vaccines for humans against infectious diseases such as *Haemophilus influenza* type b, Diphtheria, Tetanus, acellular Pertussis, Meningococcus and Pneumococcus [10,11]. However, immunogenicity is impaired without the addition of adjuvant (*i.e.*, Alum). Although many potential antigens have been discovered, the lack of universal protection may be due to the inability of conventional delivery methods to elicit the immune responses appropriate for a particular infectious agent (in addition to the well-known phenomenon of antigenic variation which is a major limitation for pathogens such as HIV and *Plasmodium*). Most protein subunit vaccines primarily induce antibody-mediated humoral responses; therefore, there is an urgent need for a new delivery method that delivers antigen to elicit more potent antigen-specific cellular responses.

Utilising nanotechnology in the field of medicine has gained pace in recent years, and nano-sized materials of less than 1 µm have presented promising potential as drug and antigen delivery systems. Nanoparticles and nanocapsules can stabilise vaccine antigens and ensure delivery to intracellular compartments to increase vaccine immunogenicity, which subunit vaccines cannot achieve. Nanoparticles such as silica, liposomes and, more recently, synthetic polymer particles have been developed as vaccine/drug carriers, and many are being studied comprehensively as promising candidates. These nanoparticle vehicles have been demonstrated to be efficiently taken up by dendritic cells (DCs), the cells which control the fate of an antigen-specific immune response. These particles therefore have the ability to induce strong humoral and cellular responses.

This review provides an overview of the advances made towards next generation vaccine development, in particular using nanotechnology, and highlights the potential of a new approach for nanocapsule preparation using a templating system. It will also explore the cellular fate of nanoparticle vaccines.

## 2. Choice of Material for Nanoparticle Vaccine

A variety of materials exist from which nanoparticles can be synthesised. Some inorganic materials such as silica and iron oxide nanoparticles have shown potential as a delivery system (reviewed in [12,13,14], however their toxicity and low clearance rate from the body raises a few concerns [15,16,17,18]. Therefore, more biocompatible and biodegradable materials have gained interest as nanomedicines (for either drug or antigen delivery).

Examples of such materials are: lipid (viral envelop or phospholipids) [19,20,21,22], synthetic polymers such as poly(allylamine hydrochlroride) (PAH) [23,24,25,26], poly(acrylic acid) (PAA) and poly(methacrylic acid) (PMA) [23,27], poly(lactide-co-glycolide) (PLGA) [28,29,30,31], and polypeptides such as poly-l-lysine (PLL) [32,33], natural polymers such as chitosan [34,35,36], and protein such as albumin [37,38,39,40]. Although the formulation of nanoparticles and preparation techniques are the same for antigen and drug delivery systems, a few examples of various types of nanoparticles studied for antigen delivery are listed in Table 2.

Amongst nanoparticle delivery systems, liposomes were one of the first to be studied. Liposomes are self-assembling phospholipid bilayer micelli with an aqueous core. Liposomes can be fabricated in a multilayered structure; therefore, they can allow the encapsulation of both hydrophilic and hydrophobic antigens between different layers. There are currently two virosome vaccines, consisting of viral phospholipid membrane, approved for human use (Inflexal^®^ V and Epaxal^®^), and more traditional liposome vaccines are in different phases of clinical trials (Stimuvax and RTS,S/AS01 in Phase III) (reviewed in [41]). Several other liposomes are available for therapeutic drug delivery of anti-cancer agents and antimicrobials [42]. Several polymeric nanoparticles are also available for therapeutic use [43]. PLGA is perhaps the most studied polymer material for antigen delivery due to its biodegradability.

**Table 2:** Examples of various types of nanoparticles studied for antigen delivery.

Category	Nanoparticle Material	Size	Antigen (pathogen)	Ref.
**Inorganic (Non-degradable)**	Iron Silica	20–300 nm	MSP1 (*Plasmodium falciparum*) BSA	[14,44]
**Liposome (Non-viral lipids particle)**	Cholesterol Lipid Lipid	200 nm	Polysaccharides (*Streptococcus pneumoniae* serotype 14) VMP001 (*Plasmodium vivax*) RTS,S/AS01_B_ (*Plasmodium falciparum* CSP + hepatitis B protein hybrid)	[45,46,47]
**Virus-like particle**	Viral capsid expressed in Bacurlovirus Bacteriophage expressed in *E. coli* C41	27–60 nm	Capsid protein L1 + L2 (HPV) Capsid protein L2 (HPV)	[19,20]
**Polymeric**	Chitosan	160–1000 nm	Hepatitis B	[30,48,49,50,51,52]
	PLGA		Ovalbumin	
	PLGA		Tetanus toxoid	
	PVPON_Alk_		Ovalbumin	
	γ-PGA		gp120 (HIV-1)	

## 3. Nanoparticle as an Efficient Antigen Delivery System

Delivering antigens in particulate form offers several advantages over soluble antigens. Antigens are encapsulated within the nanocapsules to provide protection from extracellular protease degradation and prolong their circulation in the system. Antigens can also be adsorbed on the surface of nanoparticles, sometimes in combination with adjuvants (e.g., pathogen-associated molecular patterns (PAMPs)), which allows direct interaction of the antigens with immune cell surface receptors (*i.e.*, Toll-like receptors (TLRs)) (discussed in Section 6). The particulate form also facilitates more efficient cellular uptake by antigen presenting cells (APCs), therefore making them capable of inducing potent antigen-specific humoral and, more importantly, cellular responses by promoting a higher level of cross-presentation.

### 3.1. Trafficking of Antigen to the Lymph Nodes

The efficacy of a nanoparticle vaccine depends on the interaction of the particles with DCs as they are the most important cells involved in initiating an immune response. DCs are capable of inducing primary responses to infection, and the fate of subsequent responses depends on how they respond to that particular pathogen. They are also the most potent antigen processing cells which present antigen from pathogens for subsequent activation of two major T cell types.

Different DC subsets comprise distinct phenotypes and exhibit a variety of functional properties [53]. Both resident and migratory DCs express high levels of cluster-of-differentiation (CD)11c, but resident DCs are characterised by the expression of CD8a (thus CD8^+^ or CD8^−^ DC), while migratory DCs are characterised by the expression of CD11b (thus CD11b^+^ or CD11b^−^ DC), along with expression of CD205 (DEC205) in all populations [54,55]. DEC205^+^ expressed on migratory DCs, especially on the dermal DCs, is an endocytosis-mediating receptor involved in exogenous antigen uptake, and which targets the late endosome or lysosome for MHC class II presentation [56]. It is often that the migratory type of conventional DCs play an essential role in antigen presentation and immune induction against pathogens.

Once the migratory DC captures the antigen, it will migrate out from the peripheral tissues into the draining lymph nodes through afferent lymph vessels [57]. It will then complete its journey for the interaction and activation of naïve T cells. The ability of migratory DC to transfer internalised antigens to the lymph node resident DC has also been documented [58,59], instead of directly presenting antigen themselves and priming T cells in lymph nodes. After exposure to stimuli, and following the maturation process, DCs lose the ability to further endocytose and process newly countered antigens [60]. DC migration is attributed to the changes in the expression level of surface adhesion molecules and cytoskeleton modification during the maturation process [61,62]. Therefore, the activation of T cells is limited by the presentation of specific antigen internalised by APCs prior to maturation.

While larger particles must rely on the migratory DCs for the uptake at the administration site to be transported to lymph nodes [63], some of the particulate delivery system using much smaller particles can directly target the resident DCs for antigen uptake instead of relying on capture by the migratory DCs. Nanoparticle trafficking to draining lymph nodes has been observed, and this is thought to depend on particle size [63,64,65]. Nanoparticles of less than 200 nm in diameter can freely drain to the lymph nodes spontaneously by leaving the interstitial space and being transported via the interstitial flow [66], with 20 nm range being the most suitable nanoparticle size to be transported [65,67]. 

Reddy *et al.* assessed the feasibility of nanoparticle transport to the lymph nodes using 25 nm and 100 nm polypropylene sulphide nanoparticles loaded with ovalbumin in mice [67]. They found that the 25 nm nanoparticles reached the lymph nodes much more efficiently than the 100 nm nanoparticles following intradermal injection. The subsequently induced ovalbumin-specific IgG level induced by the 25 nm nanoparticles was due to an initiation of cellular immunity by CD4^+^ T cells, and was equivalent to that of soluble ovalbumin formulated with adjuvant, whereas the level induced by the 100 nm particles was significantly lower. This study was followed by Manolova *et al.* who demonstrated that nanoparticles injected intracutaneously are trafficked to the draining lymph nodes in less than 2 h, while nano- and micro-particles of 200 nm to 2 μm, respectively, require 8 h to enter the subcapsular sinus [63]. Another study demonstrated that particles in the viral range (40–50 nm) are an optimal size to be trafficked to the draining lymph nodes with larger numbers of cells with localised beads compared to 20 nm nor 1 µm beads at both 48 h and 14 days [64]. These nano beads were found to be localised in mature DCs expressing DEC205^+^, CD40^+^, and CD86^+^ which are primary cells found in the subcutaneous region, while 1 µm beads were found in F4/80^+^, CD80^+^ cell, described as macrophage-like subsets of APC. The authors suggested that nano beads induce the activation of DCs and cause migration from dermal sites. These findings show that smaller nanoparticles have the potential to directly target lymph nodes, and this DC-independent trafficking can decrease the time to antigen presentation [68].

### 3.2. Nanoparticle-Induced Immunity

Antigen delivered by particles are internalised via various endocytic pathways depending on their size. Generally, particles larger than 1 µm, *i.e.*, the size range of a bacterial pathogen, are internalised via phagocytosis. Smaller antigens (~1 µm) are internalised via macropinocytosis, and even smaller antigens (virus sized) are internalised by receptor-mediated clathrin endocytosis (~120 nm) [69], clathrin-independent and caveolin-independent endocytosis (~90 nm) [70], or caveolae-mediated lipid rafts [71]. As different sizes of nanoparticles can be endocytosed by various pathways, it is therefore possible that the entry pathway is mediating the intracellular fate of antigen processing and subsequent T cell activation.

While the size of nanoparticles can induce different modes of cellular uptake, the differences in size may not corroborate to the type of immune response induced. Several studies demonstrated different outcomes of immune response induced by various sizes of nanoparticles. One study showed that nano beads of 40–49 nm could activate CD4^+^ T cells and induce Th1 biased cytokine secretion, while nano beads of 93–101 nm induced Th2-biased cytokine secretion following immunisation in mice [72]. In another study, polystyrene beads of 40–50 nm were able to induce cellular responses by activating CD8^+^ T cells with IFN-γ production, probably due to the nano beads being trafficked to the draining lymph nodes and activating a particular subset of DC that is efficient at cross-presentation [64]. Nanoparticle induced cellular and humoral responses were also shown to be translated in larger animals, with high induction of antigen-specific Th1-biased responses with IFN-γ production when polystyrene beads of 48 nm covalently bound to antigen were used in sheep [73]. Nano beads of this size used in the sheep study were also efficiently trafficked to the sheep’s draining lymph nodes [74], agreeing with the observations made in mouse studies. This suggested that the smaller nanoparticles can elicit both humoral and cellular responses by activating both CD4^+^ and CD8^+^ T cells.

On the contrary, it was shown that the nanoparticles of much larger size (350 nm–1 μm) could also be cross-presented while inducing a robust Th1 response with predominant IFN-γ production by priming CD4^+^ T cells [46,75]. When 300 nm PLGA nanoparticles were used for mouse vaccination, they however showed the highest internalisation by immature DCs and its activation, and also generated higher antigen-specific cytotoxic T cell (CTL) responses in mice compared to larger microparticles (1, 7, and 17 µm) [76]. This indicates that nanoparticles are superior to microparticles in inducing cellular responses.

Particulate antigens have been documented to enter the cross-presentation pathway in DCs in many studies [30,31,75,77,78,79,80,81]. For example, cross-presentation of antigen was evident in one mouse study by the potent induction of CTL responses after intranasal immunisation using 250–300 nm antigen encapsulating γ-PGA nanoparticles [30]. Ovalbumin delivered by PLGA microparticles larger than 1 µm could also induce DCs to present the MHC class I-specific ovalbumin peptide to T cells [31]. Furthermore, nanoparticles in the larger size range could also be presented via the cross-presentation pathway in non-competent APCs (B cells) [46].

What determines the magnitude of cross-presentation to be induced is not fully understood. However, the endocytic pathway in which the exogenous antigen by internalised can greatly affect subsequent intracellular processing, and hence the presentation pathway [82]. This is due to the type of endocytic vesicles formed during internalisation. It was demonstrated that the mannose receptor supplied an early endosome committed to the cross-presentation on MHC class I molecules, while the scavenger receptor delivered the antigen more towards presentation on MHC class II molecules [83]. It has also been suggested that the cytosolic pathway is the primary pathway for efficient cross-presentation [84]. The endosomal compartment is also highly acidic and internalised antigens are rapidly destroyed. This environment in the endosome might not allow sufficient time for the antigen to escape into the cytosol for cross-presentation [85]. However, encapsulation of antigen in a nanocapsule was found to maintain the prolonged release of antigen within the endosome, possibly increasing the chance of antigen to escape into the cytosol [46]. This indeed resulted in an increase in the cross-presentation of antigen. When ovalbumin-encapsulating nanoparticles were given to mice subcutaneously, it induced a significantly higher level of INF-γ than those immunised with ovalbumin-nanoparticles simply suspended together, or adjuvanted ovalbumin [86]. Harvested splenocytes were re-stimulated with CD8^+^ T cell-specific ovalbumin peptides thus confirming that the cytokine production was indeed by antigen-specific cellular responses induced by the vaccine. On the contrary, a higher level of antigen-specific IgG production was induced by the ovalbumin-nanoparticle suspension, suggesting that the higher amount of ovalbumin retained in the endosome lead to antigen presentation by the MHC class II pathway. This finding not only concluded that the encapsulation of antigen is more efficient in promoting antigen escape into cytosol, but also suggested that the type of immune response is controllable by the modified vaccine formulation using nanoparticles.

Differences in the immune responses induced by nanoparticles may not only be limited to size, but may also depend on the density of nanoparticles (solid beads or capsules), the adjuvant effects of the materials and the administration sites for the vaccination [87]. The mechanism of uptake and intracellular processing of nanoparticles may also be influenced by the surface charge or overall ζ-potential, sequence or density of polymer/antigen matrices, and other formulation properties [75]. One study demonstrated that a fusion-activated virosome internalised by receptor-mediated endocytosis actively fused with acidified endosomal membrane to release the antigen directly into the cytosol [88]. Aforementioned γ-PGA nanoparticles encapsulating antigen were taken up by DCs and found to co-localise in the same area as the soluble antigen [30]. The author suggested that the high level of cross-presentation induced by nanoparticles might be due to the amphiphilic nature of nanoparticles, aiding the antigen with endo-lysosomal escape into cytosol. This finding was supported with the observation made when hydrophobic amino acid side chains were grafted onto the same particles, and a significant increase in the membrane disruption was observed at endosomal pH (measured by the haemolytic activity on blood cell membrane) [89]. Furthermore, cationic nanoparticles comprised of PLGA can induce disruption of endosomal membranes due to cationisation from gradual acidification [90,91]. Similarly, antigen encapsulated in PLGA nanoparticles increased the level of cross-presentation by remaining longer in the endosome, and increasing the amount of antigen escape into the cytosol [46]. The level of antigen cross-presented and also the duration of presentation were also higher than for soluble antigen. Encapsulation of antigen was also shown to be more efficient than the antigen delivered on the surface of a solid particle. Encapsulation of soluble antigen can possibly protect it from endosomal protease degradation, while assisting the slow release of antigen over prolonged time, to be processed by the cross-presentation pathway. The size of nanoparticles encapsulating the antigen also has an effect on the rate of antigen degradation in endosome. γ-PGA of 40 nm and 200 nm encapsulating ovalbumin were compared for intracellular ovalbumin degradation and it was reported that the ovalbumin in smaller particles had a slower degradation rate than when encapsulated in larger particles [92]. The author suggested that this disparity in the degradation rate might be due to the nanoparticles being localised in different sites of endo/lysosome, or it could be due to the differences in polymer density used in the nanoparticles. Antigens remaining in the endosome can continue to be processed by the MHC class II pathway.

Various antigen-nanoparticles vaccine formulations can elicit different serum IgG responses. More specifically, vaccine formulations of antigen alone, antigen-encapsulating PLGA nanoparticles (~590 nm), antigen suspended together with PLGA nanoparticles (blank particles of ~480 nm), and the combination of encapsulating nanoparticles and antigen-nanoparticles suspension formulations were used to compare *in vivo* responses in a mouse study [29]. After 14, 28 and 38 days post i.m. immunisation, the antigen specific-IgG titre elicited by the combined formulation was the highest amongst the groups. Although a high level of IgG1 and IgG2a was elicited by the antigen-encapsulating nanoparticles, the combined formulation was far superior to of all formulations tested, also including both Th1 and Th2-biased cytokine production and follicular CD4^+^ T cells in lymph nodes that assist B cell maturation [93]. The depot effect of the encapsulating nanoparticles in the combined formulation probably enabled retention of the antigen at the administration site, as it was detected up to seven days post injection, while soluble antigen dissipated within 6 h. This study showed that the depot effect of nanoparticles, combined with fast drainage of soluble antigen to lymph nodes, could provide an immediate and long-term stimulation for both B cell and T cell activation.

In summary, most of the studies demonstrated that potent cellular and humoral responses can be elicited with antigen in a particulate form, significantly higher than soluble antigen, with increased production and class switching of IgG1 and IgG2a by Th2 and Th1 cytokine secretion, respectively [94,95,96]. Targeting the cross-presentation pathway for the delivery of exogenous antigen is also the key to develop effective vaccines against tumours, intracellular parasites, intracellular bacteria and some viruses [6,97].

## 4. Disadvantages of Some Preparation Methods

Organic particulate carrier systems are available in a wide variety of materials including synthetic polymers, synthetic polypeptides, polysaccharides, phospholipids and proteins, which are synthesised through various techniques. While the approval of some nanoparticles for commercial vaccines demonstrated that the particulate delivery system is biocompatible, effective and marketable, the variability caused by the manufacturing methods adds a strict limitation to approval and success [98].

Firstly, this is largely due to the requirements of harsh chemical treatment and physical stress used in the preparation process which causes degradation or possible contamination with an organic solvent (e.g., chloroform, methanol, dichloromethane) [99]. A frequently used method for encapsulating antigen is an emulsification technique, which requires the use of an organic solvent to create a water-in-oil-in-water (w/o/w) emulsion of nanoparticles. This is followed by stabilisation of the nanoparticle-antigen complex with either chemical or thermal treatment [31,100,101,102].

The main issue with this encapsulation technique is the organic solvents used to dissolve polymers and lipids, as inadequate removal of surfactant and organic solvents can result in toxicity [38]. The denaturation of antigens has also been observed [103,104], due to the solvents and sheer stress or high temperature used in the process. Furthermore, efficiency of antigen entrapment by the encapsulation method is very low, however, this method is still widely used for PLGA nanoparticles [105].

The cationic nature of liposomes (lipid particles) and polysomes (synthetic polymer particles) allows adsorption of negatively charged antigens (*i.e.*, protein, DNA), and increases electrostatic interactions between the anionic cell membrane to facilitate better uptake [106,107]. However, their cytotoxic effects add a limitation to the administration dose [108]. Nanoparticles exhibiting a strong positive electrostatic charge have greater toxic effects than strongly anionic nanoparticles, and can lead to cell death depending on the strength of the charge [31,106]. A decrease in antigen presentation and reduction in metabolic activity are also associated with the cytotoxicity of strongly cationic particles [106,109]. The net positive charge of a nanoparticle surface can also lead to rapid agglomeration and binding to serum proteins and erythrocytes [110,111]. This may cause particle clearance from tissues where APCs reside, thus hindering the process of antigen uptake [112]. Cationic nanoparticles may also cause inflammation mediated by the reactive oxygen species generated through the burst (rupture) of liposomes and PLGA particles, and occurs upon releasing the internal contents of particles [113]. Cationic nanoparticles may also cause damage to the intracellular microenvironment from the production of acid from polymer hydrolysis during degradation, as is the case with PLGA nanoparticles [90]. To overcome such issues, the addition of poly(ethylene glycol) (PEG), other hydrophilic polymers, Mg(OH)_2_ or surfactants to coat the surface of cationic nanoparticles is utilised [114,115]. While PEG-coating is often used for liposome and polymer nanoparticles currently on the market or in clinical trials for both drug and vaccine delivery, this however reduces the efficiency of cellular uptake and inhibits endosomal escape into cytosol [116].

Other commonly used techniques to fabricate nanoparticles include: self-assembly [117,118,119], inkjets [120], desolvation [38,121,122], and nanoparticle albumin-bound (nab-) technology for albumin [123,124]. While some of these methods do not require the use of organic solvents nor physical stress, the resulting nanoparticles often have high polydispersity in regard to size.

This draws attention to the importance of employing a simpler preparation method, which enables the fabrication of nanoparticles with increased homogeneity and stability. In particular, methods to utilise fewer materials for preparation by employing less cationic or amphiphilic materials to reduce cytotoxicity is a preferred option to fabricate a safe and effective vaccine delivery platform.

## 5. Novel Approach—Nanocapsule Assembly Using Templates

A templating method has also been employed in the synthesis of polymer nanocapsules [125,126]. This approach uses adsorption of polymer to the surface of monodispersed silica nanoparticles by electrostatic interaction, followed by the subsequent removal of the nanoparticles to form a hollow polymer nanocapsule [31,35]. This sacrificial template system can provide greater mechanical stability to capsule formation during the preparation process [127].The templating method allows the fabrication of nanocapsules that overcome some of the issues associated with other preparation techniques.

In the non-templating system, the broad size distribution of synthesised nanocapsules can range from nanometres to micrometres. However, the use of a monodispersed, colloidal nanoparticle allows the synthesis of nanocapsules within a narrow distribution [25]. A sacrificial template can also provide greater mechanical stability to the capsule formation during the preparation process [127]. Moreover, this method does not rely on the use of organic solvents and surfactants required for the fabrication of liposome or polysome nanocapsules, as chemically cross-linking the adsorbed antigens and polymers eliminates the need for antigen encapsulation by emulsification. Finally, the properties of nanoparticle templates can provide precise control over their size, composition, colloidal stability, permeability and surface functionalisation [128].

**Figure 1:**
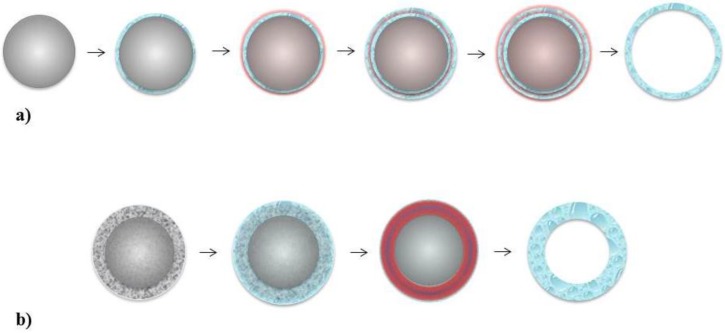
Schematic representation of (**a**) LbL assembly process, and (**b**) single step assembly with SC/MS silica template. In LbL assembly, a solid silica nanoparticle (grey) is coated with antigen (blue) by electrostatic interaction, followed by alternate deposition of polymers to create multiple layers by chemical cross-linking (red). The silica nanoparticle template is removed, leaving an antigen containing nanocapsule. In single step assembly, antigen or polymer is infiltrated into the mesoporous matrix (blue) of SC/MS silica nanoparticles (grey with mesh layer). Antigen or polymer is subsequently cross-linked (red), leaving hollow antigen-containing nanocapsules.

There are several techniques to synthesise nanocapsules which utilise the templating method. The most commonly used technique is the layer-by-layer (LbL) assembly approach, which uses solid spherical nanoparticles as a template (shown in Figure 1a). More recently, a single step assembly approach employing a solid core/mesoporous shell (SC/MS) nanoparticle has been demonstrated (shown in Figure 1b). Both methods provide properties that are superior to the non-templating methods, however, use of SC/MS nanoparticles over the LbL assembly approach offers several distinct advantages [25]. A comparison of the two different techniques is summarised below.

### 5.1. Layer-by-Layer

In LbL assembly, a solid spherical nanoparticle, is used as a sacrificial template to sequentially deposit multiple layers of polymers and antigen. The layered complex is then chemically cross-linked to immobilise the bound material, and then the template is removed [78]. The polymers and antigens usually consist of opposing charge and are adsorbed by their electrostatic force or similar interactions (*i.e.*, van der Waal) [129]. The multilayer structure enables the combination of materials with different properties to be adsorbed in the one structure. The LbL approach has a greater advantage over non-templating techniques due to its fine control over size and composition of the nanocapsules synthesised. This is particularly important in the production of a vaccine as it minimises variability by allowing for delivery of an antigen in a measurable dose.

Polymer nanocapsules assembled with this method have shown their potential for antigen delivery. It was demonstrated that nanocapsules synthesised with a variety of polymers could bind and be internalised by blood APCs *in vitro*, and HIV peptide-loaded nanocapsules efficiently induced MHC class I presentation, and activated CD8^+^ T cells [128,130]. The efficiency of nanocapsules assembled by the LbL approach was further assessed *in vivo* as an antigen delivery system, and showed that ovalbumin-loaded thiol-modified PMA (PMA_SH_) nanocapsules could induce higher levels of humoral and cellular responses than when ovalbumin was administered alone [78].

### 5.2. Single Step Assembly

Despite the advantages that LbL assembly can offer, sequential adsorption of materials is labour intensive and time and material-consuming. The deposition of materials is also driven by electrostatic forces or similar interaction of molecules, therefore, the overall thickness of a single layer is very limited within these transient interactions [25]. This results in lower antigen loading per nanocapsule, which does not alleviate the problem associated with some of the other preparation techniques. Furthermore, this assembly approach does not result in a single component of material (*i.e.*, polymer or antigen) in the nanocapsule structure. Therefore, the control over nanocapsule behaviour *in vivo* becomes more complicated.

To overcome the limitations of LbL assembly, different types of nanoparticles can be employed as a template. A mesoporous sphere is a spherical nanoparticle with a porous structure throughout. The adsorption of polymer or antigen is performed using the same principle as the LbL method, with molecules adsorbed onto the surface by the electrostatic interaction. However, molecule adsorption can be performed in a single step by infiltrating the material into the mesoporous matrix. The infiltrated material is then chemically cross-linked for immobilisation, then the template is removed to form a porous nanoparticle/capsule [131]. As the specific surface area is significantly larger with the intercalating pores, this allows a larger capacity of molecules to be loaded per nanoparticle, in a single step. A mesoporous sphere has been used to load water-insoluble compounds [24] and various types of enzymes [131,132].

Another nanoparticle in this class is the SC/MS nanoparticles. The fabrication method of this type of nanoparticle has been defined since the primary method was established by Büchel and colleagues in 1998. They utilised a highly monodispersed solid silica core as the inner base [133], to build a highly robust mesoporous shell surrounding the core [134]. Recently, SC/MS nanoparticles were employed as a template to synthesise polymer capsules. This approach was demonstrated for the first time and produced polymer nanocapsules which were highly stable at physiological pH, yet amenable to degradation by intracellular protease [25]. In addition, the nanocapsules consisted of only a single component with a thick capsule wall resulting in higher loading of materials per nanocapsule compared to the nanocapsules prepared by LbL assembly. Furthermore, the structure of the polymer nanocapsule synthesised using this system was highly homogeneous, and deformable. Flexibility in structure is an advantage and it has been shown previously that a deformable structure like a liposome can cross the endothelium fenestration, while rigid particles of the same diameter could not pass through [135]. The properties of the mesoporous layer in SC/MS nanoparticles are controllable, thus the thickness of the capsule wall and porosity can be tailored to fit the infiltrating moiety [35]. Most importantly, the fabrication of mesoporous silica nanoparticles is very simple, scalable and cost-effective [13].

## 6. Adjuvant

Improving the immunogenicity of a vaccine can be achieved by the co-delivery of stimulating molecules. Molecules such as PAMPs can induce more efficient uptake of a vaccine and ensure the activation of DCs. Adjuvants are defined compounds that are added to the vaccine to enhance antigen-specific immune responses. Adjuvants approved for human used are aluminium hydroxide mineral salts (Alum), MF59^®^, virus-like particles, cholera toxin, and MPL^®^ (glycoprotein) [136]. However, conventional adjuvants such as Alum have been historically shown to elicit strong humoral responses with weaker cellular responses. Alum is also available in particulate form made of aluminium hydroxide in micron-size, however aggregation is the major obstacle for physical characterization (*i.e.*, DLS) and *in vitro* evaluation (*i.e.*, cellular uptake) (reviewed in [137]). More potent adjuvants are available, however, their use in human is limited due to their high toxicity. The optimal balance between the toxicity of adjuvant and combination with the vaccine must be carefully considered.

An important feature of a particulate vaccine is the ability to directly conjugate an additional molecule on the nanoparticle surface. The conjugation of PAMPs with either protein or a DNA sequence can be achieved by adsorption by electrostatic interactions and chemical cross-linking [138,139]. A variety of PAMPs have been studied as co-delivery molecules in a particulate delivery system, which specifically target the matching TLRs (reviewed in [140]), however, the most commonly studied element is the unmethylated CpG motif. The CpG motif is rich in cytosine and guanine, and is recognised by TLR9, which is primarily expressed in the endosome of plasmacytoid DCs in human [141]. The use of the CpG motif in a particulate delivery system to induce cellular responses was demonstrated using PLGA nanoparticles surface-modified with the CpG motif. This combination elicited humoral, as well as cellular responses, and provided protection against live viral infection [142]. Interestingly, encapsulation of the CpG motif has also been shown to increase the cellular response much higher than the soluble antigen formulated with the CpG motif in solution, encapsulated antigen without CpG motif, and encapsulated antigen delivered with CpG in solution. The CpG motif co-encapsulated with tetanus toxoid in PLGA nanocapsules increased the level of IFN-γ, IgG2b and IgG3 by Th1-biased immune response, as well as IgG1 by Th2-biased immune response [47].

A class of nanoparticles that show promise, particularly when delivering PAMPs, are calcium phosphate-based particles. Another example of a PAMP is flagellin, a major structural component of the bacterial flagella filament. Flagellin is recognised by TLR5 expressed in monocytes, epithelial cells and immature DCs including Langerhans cells of the skin and mucosa [143,144], and induces cell activation and release of proinflammatory chemokines, as well as activating the NF-κB pathway [145,146,147]. Due to its ability to induce innate immune responses, flagellin is considered to be an attractive candidate as a vaccine adjuvant. Use of recombinant *Salmonella typhimurium* flagellin in soluble form as an adjuvant in an H5N1 recombinant haemagglutinin subunit vaccine showed an increase in mucosal IgA and serum IgG in immunised mice [148]. Flagellin can also directly stimulate human peripheral blood CD4^+^ T cells to up-regulate the production of IL-8 and IFN-γ, and increase T cell proliferation by secretion of IL-2 [149]. It also has an effect on the memory T cells, especially the effector memory T cells and it can up-regulate IFN-γ production and proliferation more effectively than naïve T cells [149]. Similarly, the effect of flagellin was evident and magnified when flagellin was conjugated to calcium phosphate nanoparticles and used for immunisation of mice. It induced a higher level of IL-6, a proinflammatory cytokine, in serum than soluble flagellin given without nanoparticles [150]. In the same study, it was observed that the flagellin functionalised nanoparticles and also up-regulated the production of other proinflammatory and potent immune modulating cytokines such as IL-8 and IL-1β in human intestinal epithelial cells (Caco-2) and *ex vivo* macrophages, respectively. This agrees with the fact that TLR5 is expressed in various cells, thus highlighting the inflammatory response as a first defense mechanism of innate immune responses. Interestingly, this study observed that the significant level of IL-1β was also up-regulated by unconjugated nanoparticles and inactive flagellin-conjugated nanoparticles, suggesting that calcium phosphate nanoparticles (or possibly due to the crosslinking agent) can increase stimulation nonetheless [150].

When calcium phosphate nanoparticles are coupled with CpG and antigen, it can lead to a potent activation of DCs and subsequently T cells; furthermore, providing protection against infection. CpG functionalised calcium phosphate nanoparticles encapsulating MHC class II-restricted HA peptide of influenza virus have not only been shown to be efficiently taken up by DCs but also to induce strong antigen specific CD8^+^ T cell responses without diminishing the effect on CD4^+^ T cells [151]. Although the induction of both CD4^+^ and CD8^+^ T cells was also observed in the cells stimulated *in vitro* with the soluble CpG and antigen mixture, the percentage of CD8^+^ T cells detected in the spleen of immunised mouse was significantly higher with mouse immunised with particulate vaccine than those with soluble CpG and HA peptide mixture (both i.p. injection). The level of INF-γ production by both CD4^+^ and CD8^+^ T cells was also much higher in the particulate vaccine groups highlighting that the cross-presentation is indeed inducible at a higher level by the use of nanoparticles.

Certain subsets of DC can cross-present more efficiently than others. CD8^+^ resident DCs in secondary lymphoid organs have the ability to cross-present [55], and CD103^+^ migratory DCs are also known to cross-present exogenous viral antigens [152,153], and apoptotic-cell antigens [154], to CD8^+^ T cells. The stimuli released upon capturing the nanoparticle can also impact on cross-presentation. Pooley *et al.* reported an effect of LPS on cross-presentation, as it increased the ability of CD4^+^ DC to present antigen to CD8^+^ T cells, when these DC subsets were often found to primarily prime CD4^+^ T cells [155]. The ability of CD8^+^ T cells to cross-present was not altered by the presence of LPS.

These findings indicate that lower amounts of potentially toxic adjuvant can be used in conjunction with nanocapsules to induce more potent humoral and cellular immunity.

Coupling of TLR ligands and PAMPs is not the only option for targeting DCs. DCs express various other surface pattern recognition receptors including C-type lectin receptors. C-type lectin receptors comprise various types of carbohydrate binding domains and are known to be involved in the phagocytic mechanism [156]. One example of a C-type lectin receptor is the mannose receptor (CD206), which recognises mannosylated glycoproteins. Glycosylated antigen was shown to be taken up efficiently by mannose receptor mediated endocytosis and also found to be localised in the MHC class II compartment [157], and di-mannose functionalised nanoparticles could activate DCs [158], however, they may require further cativation to promote phagocytosis [159]. The surface of nanoparticles can be functionalised with DC-specific antibodies. Mintern *et al.* demonstrated that the CD11c and DEC205^+^ DC subsets could be targeted by functionalisation of nanoparticles with monoclonal antibodies [48], therefore targeting particular subsets of DC (subsets of DC are discussed in Section 3.1). Anti-DEC205 conjugated PLGA nanoparticles could induce high levels of IFN-γ by antigen-specific CD8^+^ T cells indicating efficient cross-presentation, most likely due to higher intracellular uptake of nanoparticles [28].

## 7. Conclusions

It is clearly evident that new types of vaccines are required to prevent pathogenic infections that are currently unpreventable. Nanoparticles in various formulations have shown some considerable advantages over subunit vaccines in many studies. While the nanoparticles can target DC and are trafficked to draining lymph nodes for faster immune response induction, their ability to induce an antigen-specific cellular response is the key to next generation vaccines. Encapsulation of antigen and coupling of adjuvant opens up many possibilities to cater for any pathogenic infection. These small particles packed full of wonders hold the key to the success of our future vaccines.
